# Awareness and Perceptions of “Age-Friendly”: Analyzing Survey Results from Voices in the United States

**DOI:** 10.3390/geriatrics8030058

**Published:** 2023-05-28

**Authors:** Lauren Dunning, Diane Ty, Priyanka Shah, Mac McDermott

**Affiliations:** Center for the Future of Aging, Milken Institute, Santa Monica, CA 90401, USA; dty@milkeninstitute.org (D.T.); pshah@milkeninstitute.org (P.S.); mmcdermott@milkeninstitute.org (M.M.)

**Keywords:** age-friendly, aging, communities, health systems, awareness, perceptions

## Abstract

The term “age-friendly” is widely used to describe cities, communities, health systems, and other environments. However, little is known about how this is interpreted or what the term means to the public. To investigate the public’s familiarity with the term and gain insights into its relevance to older adults, we utilized data generated by a survey of 1000+ adults aged 40 and above. We employed a 10-question survey, distributed online in the US from 8 to 17 March 2023 via a third-party vendor, that captured awareness and perceptions of age-friendly designations by exploring awareness of the term, contextual understanding, and influence on decision making. The resultant aggregate data was analyzed using Microsoft Excel and straightforward summary statistical analyses. The majority of respondents (81%) were aware of the term “age-friendly.” Older adults (ages 65+) lagged in the self-described extreme or moderate level of awareness compared to adults aged 40–64. In the surveyed population, the term “age-friendly” was most often understood to apply to communities (57%), followed by health systems (41%) and cities (25%). Most people believed “age-friendly” refers to all ages, even though age-friendly health systems are designed to meet the unique needs of older adults. These survey results provide the age-friendly ecosystem field with insights into the awareness and perceptions of the term “age-friendly,” highlighting opportunities to bolster understanding.

## 1. Introduction

With the population aged 65 and over projected to nearly double by 2050, the US is facing a significant demographic shift. Approximately 85.7 million people will be over 65, comprising around 22% of the total population [1]. This demographic shift has far-reaching implications for all sectors of society, including healthcare, housing, social services, and the economy, requiring innovative solutions to meet the needs of an aging population.

In response, age-friendly initiatives were developed and have gained significant momentum in recent years. Globally, the age-friendly movement began when the World Health Organization (WHO) first introduced the concept of age-friendly cities and communities (AFCs) in 2006 [2]. WHO defined AFCs as places that promote “active aging by optimizing opportunities for health, participation, and security in order to enhance quality of life as people age” and laid out a framework of eight key domains: outdoor spaces and buildings, transportation, housing, social participation, respect and social inclusion, civic participation and employment, communication and information, and community support and health services [3].

Spurred by initiatives such as AARP’s Network of Age-Friendly States and Communities, the age-friendly concept quickly gained traction in the US. Since 2012, more than 750 communities nationwide have committed to actively working toward becoming more age-friendly [4]. AARP defines AFCs as those with “a commitment to being more livable for people of all ages, and especially older adults” [5]. Regional studies examining AFCs have found a variety of positive impacts among older adults, including better self-rated health and fewer functional limitations and chronic health conditions [6,7,8]. Higher quality of life and well-being, increased social connectedness and community involvement, and greater perceived neighborhood safety, accessibility, and support were also found among residents of age-friendly communities [9,10,11].

Health services are a critical domain of AFCs and focused efforts aim to increase their age-friendliness, including globally through the WHO Age-Friendly Principles developed for primary care and expanded to additional settings [12,13]. In the US, the Institute for Healthcare Improvement and The John A. Hartford Foundation, in partnership with the American Hospital Association and the Catholic Health Association of the United States, launched the Age-Friendly Health Systems (AFHSs) initiative in 2017 to scale and spread efforts [14,15]. AFHSs provide high-quality care that is both effective and efficient while meeting the unique needs of older adults, improving outcomes, and reducing costs [16]. As of 2023, over 3000 health systems across the nation have joined the initiative, committing to implementing the four core elements or “4Ms”—What Matters, Medication, Mentation, and Mobility—of age-friendly care in their organizations [17]. Evaluations of the clinical impact of AFHSs have identified improvements in both care and quality [18]. 

The expanding footprint of age-friendly designations has driven inquiry into their benefits and impacts [19]. Many studies examine assessment methods for AFCs [19] and evaluate implementation outcomes [20]. Research exploring individual-level awareness of the term “age-friendly” in the context of AFCs and AFHSs is currently limited, though, and of importance to the field as aging-in-place and age-friendly community initiatives have been linked to ecological frameworks and the factors of the socio-ecological model [21,22]. The social ecological model of healthy aging, which articulates individual and social environmental factors as components of multi-level health promotion interventions, includes intrapersonal influences such as awareness and knowledge alongside community attributes and the policy environment [23,24]. Operationalizing this model, AFC initiatives often involve activities focused on building awareness among residents [25], with research on barriers and facilitators of AFCs identifying awareness-raising as a factor supporting successful implementation [20].

Studies focused on AFHSs find low awareness among the public, with only 25% of older adults and family caregivers reporting being aware of age-friendly care [26] and 60% of physicians claiming familiarity with AFHSs [27]. Given the limited understanding of the general public’s awareness of age-friendly designations and their influence on individuals, as well as the potential importance of increasing familiarity and engagement with age-friendly initiatives, we obtained third-party survey data of over 1000 individuals to address the following questions: (1) how is age-friendly understood or described by the public? (2) Does this awareness or understanding vary with the age of the population? (3) Is the term understood to apply to communities, individuals, or something more? We hypothesized that the term age-friendly would be more familiar to those over 65, understood to apply primarily to older adults, and most frequently recognized as pertaining to communities. Due to the paucity of data regarding the awareness and perceptions of AFCs among adults, we conducted this study to begin to address this critical gap in the literature. 

## 2. Materials and Methods

### 2.1. Study Population

We engaged a third-party online survey vendor to assess awareness and perceptions of the term “age-friendly” among a sample of over 1000 members of the US public aged 40 and older. The age category of 40 and older was selected because age-friendly initiatives typically focus on and are relevant to older segments of the population. By including those over age 40 and not just over age 65, perspectives of today’s older adults and the older adults of the near future who are currently in midlife were captured, which is a common approach in surveys on aging-related topics [28,29]. We selected the age brackets of 40–49, 50–64, 65–74, 75–84, and 85 and over to allow analysis of responses for different age categories. The survey respondents were randomly selected members of the vendor’s existing national panel of adults aged 40 and over, with the requirement that at least 25% of respondents were aged 65 and older.

### 2.2. Survey Design, Administration, and Analysis

The cross-sectional survey design included 10 questions, as listed in Appendix A. The survey consisted of quantitative and qualitative measures using multiple choice, Likert-like rating scales, and open-ended responses. These novel measures were informed by the literature on age-friendly designations and their characteristics [5,30]. The survey had five objectives aimed at understanding: (1) the level of general awareness of the term “age-friendly” and in what context(s); (2) the perceived understanding of the age range to which the term “age-friendly” applies; (3) the general context for “_______-friendly” designations in respondents’ minds and whether other aging-related phrases in the field are mentioned unaided; (4) the influence that “age-friendly” may have in consumer preferences and decision making; and (5) the degree to which individuals have taken action regarding their advance care planning, which is key in understanding “what matters” to individuals. “What Matters,” one of the “4Ms” in the framework guiding AFHSs, involves knowing and acting on each patient’s specific health outcome goals and care preferences [30]. At the conclusion of the survey, an additional six demographic questions were asked. 

The survey was active from 8 March 2023 to 17 March 2023, and was widely distributed online via a third-party vendor with a global audience panel of over 22 million members from diverse geographies, demographics, and backgrounds. The vendor recruited US-only participants through a variety of media channels, including social media, email campaigns, and partnerships with other market research companies. Five thousand eligible adults were invited to participate in the survey in order to reach a target quota of 1000 completed responses with at least 250 responses from adults over age 65. Once both targets were met, the survey was closed. 

One thousand and twenty-two surveys were included in the sample. The survey results were analyzed using Microsoft Excel to produce descriptive statistics. The responses are presented as frequencies and percentages.

### 2.3. Ethical Considerations

Survey participation was voluntary, and completion of the survey was considered consent to participate. Respondents received a modest monetary incentive through cash or a gift card to complete the survey. While the vendor collects 300 panel attributes for each respondent as part of its general screening process, these attributes were not shared with the research team and the data were analyzed in aggregate with no individual identifying information.

## 3. Results

### 3.1. Respondent Demographics

The respondents self-identified their age bracket, gender, race/ethnicity, geographical area, educational level, and profession/vocation. Displayed in Table 1, the respondents were predominantly self-identified as white, (over 79%), followed by Black or African American (about 10%) and Hispanic/Latino (4.5%). The sample predominantly skewed younger, with over 73% of respondents between the ages of 40–64, and was mostly female (over 60%). While geographical area was mixed, most originated from suburban areas, defined by the survey as “a cluster of properties, primarily residential, that are not densely compacted, yet located very near an urban area”.

### 3.2. Survey Findings

#### 3.2.1. The Level of General Awareness of the Term “Age-Friendly” and in What Context(s)

Based upon straightforward frequency analyses of the responses, several trends became clear. Across the survey sample, most respondents indicated they were “moderately aware” of age-friendly (31%) and perceived the general public as “somewhat aware” of age-friendly (32%). Disaggregating by demographic characteristics such as age, gender, geographical area, or sub-industry revealed additional trends. Table 2 demonstrates that adults aged 40–64 were the most likely to be at least “somewhat aware” of age-friendly, while ages 75–84 were the most likely to be “slightly” or “not at all aware.” Adults over age 85 were mostly “somewhat aware” of age-friendly. Similar trends persisted for the perception of public awareness, with adults aged 40–49 leading in perceived general awareness. Respondents aged 50–84 were the least likely to perceive the general public as “extremely aware” of age-friendly. Adults over age 85 most frequently cited the general public as “somewhat aware.”

Additionally, respondents living in an urban environment most often reported greater perceived personal and public awareness of age-friendly relative to suburban and rural respondents. Respondents working in the healthcare industry (sub-industries administration, research, patient advocacy, health systems, and health plans) were largely more personally aware of age-friendly than other industries.

**Figure 1:**
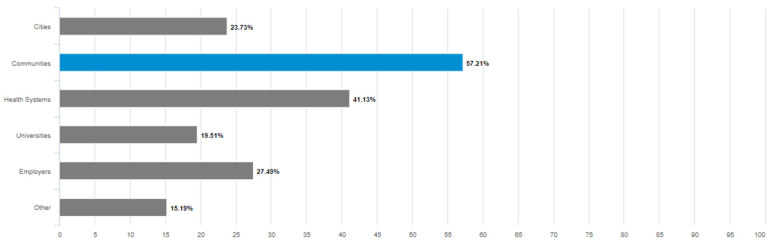
Contexts in Which the General Public Has Heard the Term “Age-Friendly” Used To Describe Something. Blue distinguishes the most frequently reported context in which respondents had heard the term "age-friendly".

Among respondents reporting any level of awareness of the term “age-friendly” other than “not at all aware,” the most frequently reported context for hearing the term “age-friendly” used to describe something was communities (over 57%), followed by health systems, employers, and cities. The least frequently recognized age-friendly context was universities (under 20%) (see Figure 1).

#### 3.2.2. The Perceived Understanding of the Age Range to Which the Term “Age-Friendly” Applies

**Figure 2:**
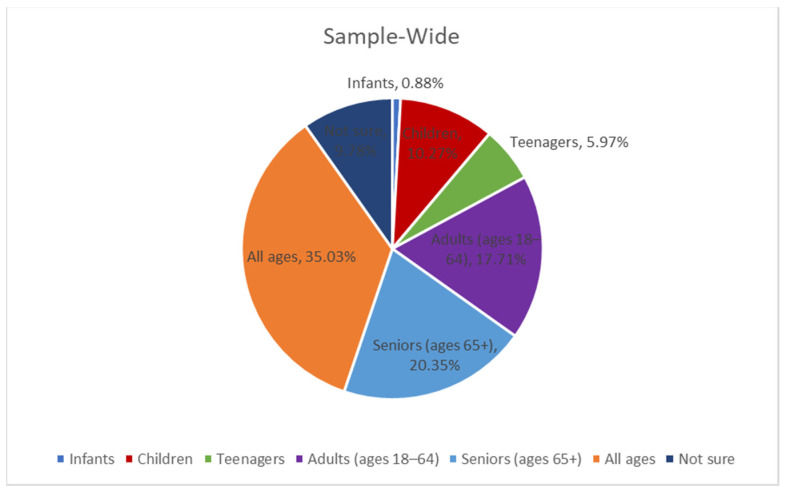
What Age Range Do You Think the Term “Age-Friendly” Applies to?

Sample-wide, only roughly 1 in 5 respondents (20.35%) indicated that the term age-friendly applies specifically to seniors (ages 65+) (Figure 2). Nearly the same percentage of respondents (17.71%) thought the term applied to individuals between the ages of 18–64. Most respondents (35.03%) said it applies to all ages. Nearly 10% were unsure.

**Figure 3:**
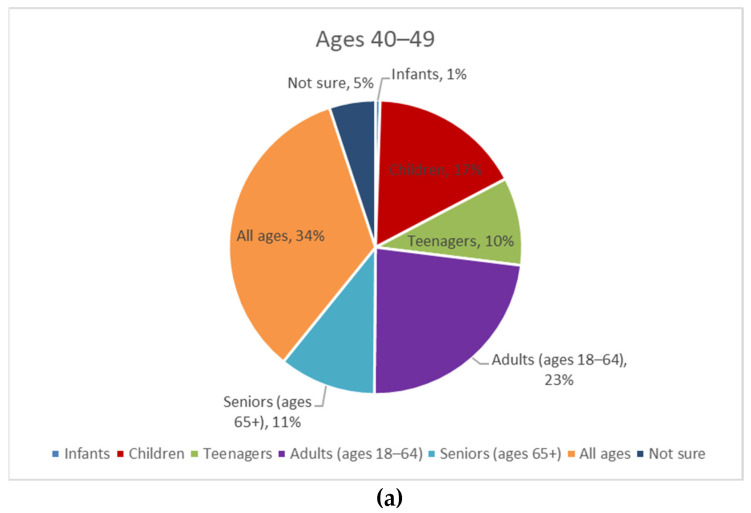

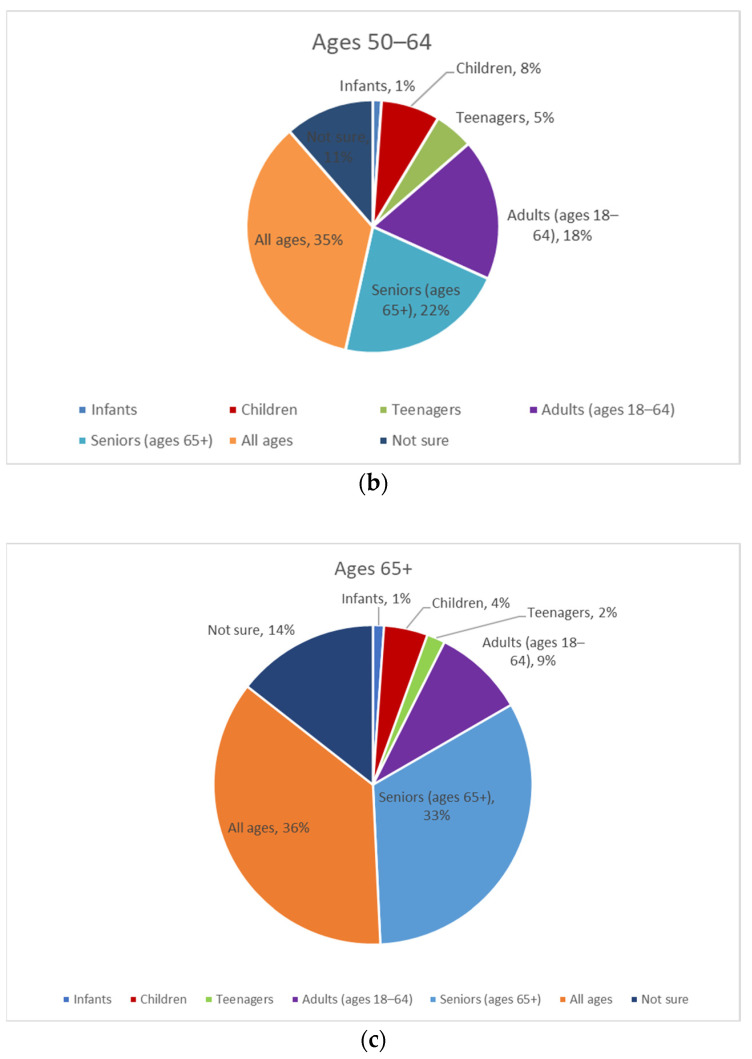
What Age Range Do You Think the Term “Age-Friendly” Applies to? (By Age: (**a**) Ages 40–49; (**b**) Ages 50–64; (**c**) Ages 65+).

An analysis by age bracket (40–49, 50–64, and 65+) showed additional findings (Figure 3). Adults aged 65+ were most likely to answer that age-friendly applies to seniors (about 33%), followed by adults aged 50–64 (22%) and adults aged 40–49 (11%). Of the three age brackets, adults aged 40–49 more frequently answered that age-friendly applies to adults aged 18–64 (23%), teenagers (10%), and children (17%) than respondents over age 50.

#### 3.2.3. The General Context for “____-Friendly Designations” in Respondents’ Minds and Whether Other Aging-Related Phrases in the Field Are Mentioned Unaided

With the age-friendly movement underway, another phrase that is designed to create a set of expectations for a supportive experience is the term “dementia-friendly.” Dementia Friendly America is a national network of communities, organizations, and individuals seeking to ensure that communities across the US are equipped to support people living with dementia and their caregivers [31]. We sought to identify if respondents are familiar with this term on an unaided basis and how the use of “____-friendly” is used and understood overall.

The respondents were thus asked if they had heard the phrase “_____-friendly” in other contexts and, if so, where or how. Only 24% had knowledge of this within other contexts. The most frequently cited examples included entertainment (e.g., movies, television, games) (66), pets (43), children and toys (41), user-friendly products or services (e.g., technology) (31), and eco-friendly (19). This open-ended question had 245 valid responses; none mentioned dementia.

The respondents were further prompted to share a word or phrase that describes the term “age-friendly.” The most frequent responses clustered around the phrase being: appropriate for all ages (153), youth (64), seniors (48), and entertainment (47). This open-ended question had 503 valid responses.

#### 3.2.4. The Influence That “Age-Friendly” May Have on Consumer Preferences and Decision Making

**Figure 4:**
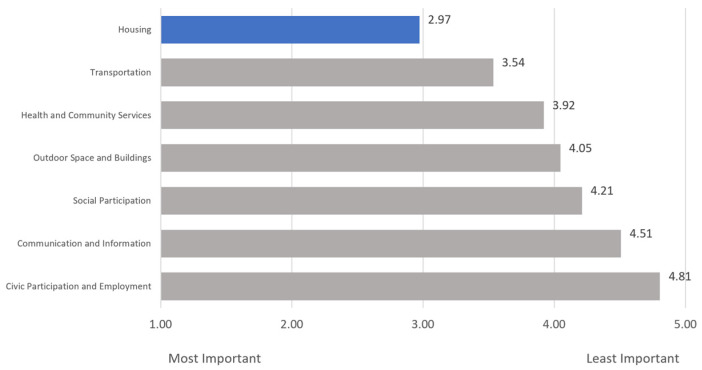
Average Rank of Age-Friendly Community Characteristic Importance (Scale: 1–7). Blue distinguishes the highest-ranked aspect of an age-friendly community identified by respondents.

The participants were asked to rank seven choices related to age-friendly communities in order of importance, with 1 being the most important and 7 being the least important. Figure 4 shows that, on average, respondents found housing, with an average rank of 2.97, to be the most important aspect of an age-friendly community, followed by transportation (3.54) and health and community services (3.92). Communication and information and civic participation and employment were ranked lowest in priority on average.

Table 3 demonstrates that respondents reporting an age-friendly designation as “extremely influential” to their decision to live in a community was highest among those aged 40–49 compared to ages 50+. Across the sample, the most frequently cited level of influence of an age-friendly community on one’s decision to live there was “somewhat influential.” However, adults aged 85+ more frequently responded that an age-friendly designation was extremely, slightly, or not at all influential. Approximately 84% of urban respondents reported that an age-friendly designation was at least somewhat important in their decision to live in a community, compared to 79% of suburban respondents and 76% of rural respondents.

#### 3.2.5. The Degree to Which Individuals Have Taken Action Regarding Their Advance Care Planning

Most respondents reported that they either had thought about or taken action on each prompt regarding advance care planning. Broadly, the respondents had most frequently taken action to ensure that they have copies or access to their health records, followed by appointing a care proxy. Additionally, respondents most frequently had thought about but had not acted on having a care plan and documenting their healthcare preferences. The respondents most frequently had not thought about taking action on appointing a care proxy.

Table 4 indicates the gender-based differences in the degree to which respondents report taking action regarding their advance care planning. Men were more likely than women to report action on each prompt—having a care plan, documentation of care preferences, appointment of a care proxy, and ensuring access to health records. Having a master’s, professional, or doctorate degree was also associated with reported higher levels of action compared to lower levels of education.

## 4. Discussion

Age-friendly ecosystems are important because they help promote well-being and quality of life for older adults, who may face challenges related to aging, such as mobility limitations, health issues, and social isolation. As age-friendly initiatives continue to spread, examining awareness of the term “age-friendly” among the public and placing it in the context of everyday life furnishes government officials, healthcare providers, policymakers, and advocates with feedback on these efforts. Further, investigating the role of individual factors within multi-level policy and system change initiatives advancing age-friendly environments can support planning and evaluation.

This study found that 8 in 10 adults aged 40 and over were at least somewhat aware of the term “age-friendly” in any context. Among adults aged 40–49, 42% report being “extremely aware” of the term “age-friendly,” while only 17% and 18% of those aged 65–74 and 75–84 reported the same. Among adults 85 and over, only 11% reported being extremely aware. These data refute our theory that the main target population of age-friendly initiatives—older adults—would have greater awareness of the term. As older adults are a primary target population for age-friendly initiatives, further inquiry into the factors contributing to their lower awareness would provide insight into advancing the impacts of AFCs and AFHSs. Middle-aged adults may be more attuned to the concept because of their role as family caregivers for their aging parents. This is consistent with previous research that found that older adult patients were more likely than caregivers to be unfamiliar with the concept of AFHSs [18].

On the association of the term “age-friendly” with specific environments and age groups, the respondents reported more frequently hearing the term “age-friendly” with communities—nearly 60%—than any other option, including health systems (41%). These findings support our hypothesis that communities are most frequently associated with being “age-friendly” given the multiple domains of AFCs that create a multitude of opportunities for touchpoints with individuals. Only one in five respondents thought the term “age-friendly” was related to older adults. However, adults 65 and over more frequently answered that the term applied to seniors (33%), compared to only a small minority of those 40–49 (11%) answering the same. This runs contrary to our expectation that most respondents would relate “age-friendly” to older adults.

The WHO promotes “age-friendly” as a framework to ensure cities and communities are working “to improve the relationships between the environment and the people who live there, regardless of their age” [32]. AARP’s efforts with the network of age-friendly states and communities [33] advocate for policies and programs that support all ages. With 750 US communities designated as “age-friendly” and growing, this may help explain why more respondents associate age-friendly with all ages instead of solely for older adults [4]. However, AFHSs, which aim to ensure every older adult receives the best care possible, have room to grow awareness of the term’s application to older adults among the general population, especially among those over 65.

Understanding how respondents independently describe the term “age-friendly” and associate “____-friendly” as a descriptor of specific contexts further spotlights the varying perceptions of age-friendly. More respondents answered “youth” than “seniors” when describing “age-friendly.” Nearly half of the valid responses for contexts of “____-friendly” focused on entertainment such as movies and television (27%) and children’s toys and games (17%), indicating that “____-friendly” for some is potentially related to the age appropriateness of content. Notably absent were mentions of the dementia-friendly movement, which is promoted in the US through Dementia Friendly America and defined as a village, town, city, or county that is informed, safe, and respectful of individuals with dementia [31]. Overall, these findings indicate that “age-friendly,” used to denote something designed for the unique needs of older adults or inclusive of older adults, may not be clearly associated by the general public with an older demographic.

Age-friendly initiatives can foster a sense of community and social connectedness among older adults and are associated with positive health outcomes [6,7,8], which can be important factors in individual decision making. To explore this in context, the survey probed the influence of “age-friendly” on the decision to live in a community. The respondents generally reported that age-friendly is somewhat influential on their decision to live in a community, with influence highest among those aged 40–49. Housing, transportation, and health and community services ranked as the most important aspects of AFCs. Taken together, these responses provide positive support for the implementation of AFCs and indicate where investments in initiatives would most closely align with preferences.

This survey also covered aspects of AFHSs in greater depth, including advance care planning. Advance care planning is needed to determine “what matters” to older adults in the framework of the evidence-based “4Ms”—the essential elements of AFHSs. Experts have recommended that Congress support legislation ensuring that every older person has an “anticipatory plan” in the event of serious illness, documentation of their care preferences and care proxies that can be shared across settings, and copies of their own health records [34]. Estimates of the number of adults that have completed an advance care directive range from one-third to nearly two-thirds [18,35].

Among survey respondents, 32% reported having a care plan in the event of a serious illness, aligning with these previous findings. Most individuals reported they had thought about but had not taken action when it comes to having a care plan in the event of a serious illness or event (45.1%), while 23.1% had not thought about it at all. The gender-based differences in taking action, where women clearly lag behind men across all four prompts, indicate the need for interventions targeting women. Women are more likely to have thought about but not taken action, which suggests that they are aware of the need but would benefit from support and incentives to convert their awareness toward completion.

Although the present study provides early insights into awareness of the term “age-friendly” and public perceptions, it has limitations. The survey sample was not nationally representative. Compared to the demographics of the US population of adults aged 40 and over, based on 2021 population estimates from the US Census Bureau, the sample differed in a number of ways. For example, there were more female respondents (over 60% vs. 52.1% in all US adults 40+), fewer Hispanic/Latino respondents (4.5% vs. 14%), and fewer older adults over the age of 65 (26.4% vs. 34.8%) [36]. As a preliminary analysis, it utilized descriptive statistics to uncover initial areas for discussion and further investigation. Inferential statistics would have helped uncover significant correlations between response variables and exploration of associations and trends. Opportunities for next steps include comparing the knowledge and awareness among groups regularly in contact with AFCs and AFHSs to those that are not and exploring the term “age-friendly” in specific cultural contexts and among more diverse communities.

## 5. Conclusions

The survey findings provide the age-friendly ecosystem field with the context in which it is vying for in the mindshare of adults over 40. Most respondents reported being aware of the term “age-friendly,” with adults aged 40–64 leading the sample in being at least “somewhat aware” and those over 65 lagging in their self-reported “extreme awareness.” Age-friendly was most often associated with cities and communities and the majority of respondents perceive the term as applying to all ages. Taken together, the results suggest that the depth and specificity of awareness of the term “age-friendly” is lacking and that it is perceived quite broadly, beyond the intended scope of some efforts. As AFHSs strive to align the care preferences of older adults with the care they receive, their target population may not clearly associate the term with an older demographic. Knowledge- and awareness-building, alongside the systemic changes that age-friendly initiatives aim to produce, can be an avenue for further examination to determine the potential contribution to outcomes and overall success.

## Figures and Tables

**Table 1:** Demographic characteristics of study population (*n* = 1022 completed responses).

Characteristic	Number of Responses (%)
**Age**	
40–49	393 (38.45%)
50–64	359 (35.13%)
65–74	196 (19.18%)
75–84	65 (6.36%)
85+	9 (0.88%)
**Gender**	
Female	616 (60.27%)
Male	406 (39.73%)
Other	0 (0%)
**Race/Ethnicity**	
Asian	27 (2.64%)
Black or African American	103 (10.08%)
Hispanic or Latino	46 (4.5%)
Middle Eastern or North African	1 (0.1%)
Multiracial or Multiethnic	5 (0.49%)
Native American or Alaska Native	19 (1.86%)
White	811 (79.35%)
Self-Describe	9 (0.88%)
Native Hawaiian or other Pacific Islander	1 (0.1%)
**Geographical Area**	
Urban	310 (30.33%)
Suburban	471 (46.09%)
Rural	241 (23.58%)

**Table 2:** Perceptions of Age-Friendly Awareness, by Age.

Prompt	Perceived Awareness (%)				
	Extremely Aware	Moderately Aware	Somewhat Aware	Slightly Aware	Not at All Aware
How would you rate your awareness of the term “age-friendly”?					
Overall	28%	31%	22%	8%	12%
40–49	42%	31%	17%	4%	6%
50–64	19%	30%	25%	9%	16%
65–74	17%	34%	23%	11%	14%
75–84	18%	26%	28%	15%	12%
85+	11%	11%	56%	11%	11%
How would you rate the general public’s awareness of the term “age-friendly”?					
Overall	14%	25%	32%	16%	12%
40–49	24%	34%	24%	10%	8%
50–64	9%	21%	36%	18%	16%
65–74	9%	20%	37%	20%	13%
75–84	3%	12%	37%	31%	17%
85+	22%	0%	67%	0%	11%

**Table 3:** Level of Influence of “Age-Friendly” on Living Community Decisions.

Prompt	Level of Influence (%)				
	Extremely Influential	Very Influential	Somewhat Influential	Slightly Influential	Not at All Influential
If you were to see or hear the term “age-friendly community,” how much would it influence your decision to live in that community?					
Overall	22%	25%	33%	9%	12%
40–49	31%	25%	27%	6%	10%
50–64	16%	25%	36%	10%	13%
65–74	15%	26%	40%	10%	10%
75–84	17%	22%	32%	11%	18%
85+	22%	11%	11%	33%	22%

**Table 4:** Action Taken Regarding Advance Care Planning (By Gender).

Prompt	Taken Action (%)		Thought About but Not Taken Action (%)		Have Not Thought About Taking Action (%)	
	Male	Female	Male	Female	Male	Female
Having a care plan in the event of a serious illness or action	39%	27%	40%	48%	21%	25%
Documentation of your health care preferences in the event you may no longer be able to make decisions for yourself	39%	28%	39%	47%	22%	25%
Appointed someone to be your care “proxy” or the person who will make care decisions for you in case you may no longer be able to do so for yourself	40%	33%	33%	39%	26%	28%
Ensuring you have copies or access to your health records	46%	41%	33%	37%	21%	22%

## Data Availability

The data that support the findings of this study are available on request from the corresponding author, L.D.

## Appendix A

Survey

We appreciate your willingness to participate and provide us with valuable insights.

We want to assure you that your responses will be kept strictly confidential and will not be identifiable to any individual. We take your privacy seriously and adhere to strict data protection guidelines. Your personal information will be kept secure and will only be used in the aggregate for research purposes Please note that this survey is designed to gather information based on your own experiences, opinions, and perspectives. We kindly ask that you refrain from consulting external sources such as search engines, reference materials, or other individuals while completing the survey. By participating in this survey, you certify that you have not used any external sources or consulted with anyone to answer the questions, and that your responses are Based solely on your own knowledge and opinions.

Thank you for taking the time to participate in our survey.

1. How would you rate your awareness of the term “age-friendly”?
  - Extremely aware
  - Moderately aware
  - Somewhat aware
  - Slightly aware
  - Not at all aware
2. How would you rate the general public’s awareness of the term “age-friendly”?
  - Extremely aware
  - Moderately aware
  - Somewhat aware
  - Slightly aware
  - Not at all aware
3. What age range do you think the term “age-friendly” applies?
  - Infants
  - Children
  - Teenagers
  - Adults (18–64)
  - Seniors (ages 65+)
  - All ages
  - Not sure
4. When you hear the term “Age-Friendly,” what is at least one word or short phrase that comes to mind that describes the term?
5. In what context have you heard the term “age-friendly” used to describe something (check all that apply)?
  - Cities
  - Communities
  - Health Systems
  - Universities
  - Employers
  - Other (please describe): ________
6. An age-friendly community is a place that adapts its services and physical structures to be more inclusive and receptive to the needs of its population to improve their quality of life as they age. Rank, in order of importance to you, factors that age-friendly communities should have.
  - **Transportation:** safe, reliable, and affordable. Easy-to-use public transportation, walking and biking paths, and rideshare options.
  - **Housing:** various options and a network of home-based care and service options to allow older adults to remain in their homes.
  - **Outdoor space and buildings**: accessible parks and green spaces, safe streets and sidewalks, and buildings that all can enjoy, including residents with mobility challenges.
  - **Social participation:** opportunities for older adults to engage in life-long learning, social, and cultural activities.
  - **Civic participation and employment:** support services for older adults to find paid jobs and volunteer opportunities.
  - **Communication and information:** systems designed to provide older adults with access to information that can help them and their care partners as they age.
  - **Health and community services:** affordable care that aligns with older adults’ health outcome goals and preferences whether care is delivered in medical or community settings or at home
7. If you were to see or hear the term “age-friendly community,” how much would it influence your decision to live in that community?
  - Extremely influential
  - Very influential
  - Somewhat influential
  - Slightly influential
  - Not at all influential
8. Which items or activities below have you thought about or taken action on in the last five years?

**Item or Activity**	**Taken Action**	**Thought About but Not Taken Action**	**Have Not Thought About Taking Action**
Having a care plan in the event of a serious illness or action			
Documentation of your health care preferences in the event you may no longer be able to make decisions for yourself			
Appointed someone to be your care “proxy” or the person who will make care decisions for you in case you may no longer be able to do so for yourself			
Ensuring you have copies or access to your health records			

9. Have you heard the phrase ___-friendly in other contexts?
  - Yes
  - No
  - Not sure
10. If yes, please name the context.

Demographics:

- What is your gender?
  ○ Female
  ○ Male
  ○ Other (please specify)
- What is your age?
  ○ 40–49
  ○ 50–64
  ○ 65–74
  ○ 75–84
  ○ 85+
- What is your race or ethnicity?
  ○ Asian
  ○ Black or African American
  ○ Hispanic or Latino
  ○ Middle Eastern or North African
  ○ Multiracial or Multiethnic
  ○ Native American or Alaska Native
  ○ White
  ○ Native Hawaiian or other Pacific Islander
  ○ Self-describe ____
- Please select your geographical area.
  ○ Urban–By definition, an urban area is the region surrounding a city. Urban areas are very developed, meaning there is a density of human structures such as houses, commercial buildings, roads, bridges, and railways. Urban area can refer to towns, cities, and suburbs.
  ○ Suburban–By definition, a suburban area is a cluster of properties, primarily residential, that are not densely compacted, yet located very near an urban area. Also referred to as the “suburbs,” these areas are often located just outside of larger metro areas but can span even further.
  ○ Rural–By definition, a rural area, often called “the country,” has a low population density and large amounts of undeveloped land.
- What is your highest level of education?
  ○ Some high school
  ○ High school
  ○ Some college
  ○ Trade/vocational/technical
  ○ Associate’s
  ○ Bachelor’s
  ○ Master’s
  ○ Professional
- Doctorate

What industry do you work in?

- Accounting
  ○ Agriculture
  ○ Apparel
  ○ Biotech
  ○ Communications
  ○ Consulting
  ○ Education
  ○ Energy
  ○ Engineering
  ○ Entertainment
  ○ Environmental
  ○ Finance
  ○ Food and Beverage
  ○ Government
  ○ Healthcare
  ○ Hospitality
  ○ Insurance
  ○ Law
  ○ Manufacturing
  ○ Media
  ○ Nonprofit
  ○ Other
  ○ Pharmaceutical
  ○ Real Estate
  ○ Retail/Shipping
  ○ Technology
  ○ Telecommunications
  ○ Transportation
  ○ Utilities
- If healthcare is selected, please specify your sub-industry identification:
  ○ Administration
  ○ Research
  ○ Biomedical
  ○ Government
  ○ Media
  ○ Patient Advocacy
  ○ Insurance
  ○ Health System
  ○ Health Plan
  ○ Provider
  ○ Philanthropy
